# Management of non-invasive tumours, benign tumours and breast cancer during the COVID-19 pandemic: recommendations based on a Latin American survey

**DOI:** 10.3332/ecancer.2020.1115

**Published:** 2020-10-06

**Authors:** G Luis Pendola, Roberto Elizalde, Pablo Sitic Vargas, José Caicedo Mallarino, Eduardo González, José Parada, Mauricio Camus, Ricardo Schwartz, Enrique Bargalló, Ruffo Freitas, Mauricio Magalhaes Costa, Vilmar Marques de Oliveira, Paula Escobar, Miguel Oller, Luis Fernando Viaña, Antonio Jurado Bambino, Gustavo Sarria, Francisco Terrier, Roger Corrales, Valeria Sanabria, Juan Carlos Rodríguez Agostini, Gonzalo Vargas Chacón, Víctor Manuel Pérez, Verónica Avilés, José Galarreta, Guillermo Laviña, Jorge Pérez Fuentes, Lía Bueso de Castellanos, Bolívar Arboleda Osorio, Herbert Castillo, Claudia Figueroa

**Affiliations:** 1 Ecuadorian Society of Breast Pathology (SEPAM Guayaquil Ecuador), Mastology Service, National Oncology Institute—Society to Fight Cancer, (CENONI) Comprehensive Oncology Centre, Guayaquil 090513, Ecuador; 2 Argentinian Society of Mastology (SAM), Pirovano Hospital Buenos Aires, Buenos Aires, Argentina; 3 Bolivian Society of Mastology, Oncology Institute of East Bolivia, Santa Cruz, Bolivia; 4 Colombian Association of Mastology, Country Hospital, Oncology Centre, Bogota, Colombia; 5 Argentinian Society of Mastology (SAM), Ángel H Roffo Oncology Institute, University of Buenos Aires, C1053 CABA, Argentina; 6 Uruguayan Society of Mastology, Image Clinic Drs. Parada, Maldonado Hospital, Dr. Elbio Rivero, Cantegril Sanatorium, Punta del Este, Maldonado, Uruguay; 7 Chilean Society of Mastology, Clinical Hospital, Catholic Pontificia University of Chile, Chile; 8 Chilean Society of Mastology, Santiago Military Hospital, Las Condes Clinic, Chile; 9 Mexican Association of Mastology, National Cancer Institute, ABC Medical Centre, Mexico; 10 Brazilian Society of Mastology, Sao Paulo, Brasil; 11 Brazilian Society of Mastology, Americas Oncology Centre, Sao Paulo, Brasil; 12 Chilean Society of Mastology, Santiago de Chile, Chile; 13 Dominican Society of Surgery, Santo Domingo, Republica Dominicana; 14 Colombian Association of Mastology, Bogota, Colombia; 15 Ecuadorian Society of Breast Pathology, Guayaquil, Ecuador; 16 Peruvian Society of Mastology, Lima, Peru; 17 Argentinian Society of Mastology, Italian de La Plata Hospital, Buenos Aires, Argentina; 18 Bolivian Society of Mastology, Santa Cruz, Bolivia; 19 Paraguayan Society of Mastology, Itauguá National Hospital, Paraguay; 20 Venezuelan Society of Mastology, Caracas, Venezuela; 21 Costa Rican Society of Mastology, San Jose, Costa Rica; 22 Mexican Association of Mastology, Mexico DF, Mexico; 23 Nicaraguan Mastology Association, Vivian Pellas Hospital Managua, Managua, Nicaragua; 24 Uruguayan Society of Mastology, Montevideo, Uruguay; 25 Honduran Society of Mastology, Mater Dei Hospital and Clinics, Tegucigalpa, Honduras; 26 Puerto Rican Society of Mastology, HIMA San Pablo Oncology Hospital, Caguas, Puerto Rico; 27 Guatemalan Society of Mastology, Ciudad de Guatemala, Guatemala; 28 El Salvador Society of Mastology, San Salvador, El Salvador

**Keywords:** coronavirus, breast cancer, survey method

## Abstract

**Introduction:**

The COVID-19 pandemic has changed health systems across the world, both in general hospitals and in oncology institutes or centres.

For cancer specialists, particularly breast cancer (BC), the COVID-19 pandemic represents a combination of challenges since the hospital resources and staff have become more limited; this has obliged oncology specialists to seek a consensus and establish which patients with BC require more urgent attention and which patients can wait until there is a better control of this pandemic. The health system in Latin America has some special characteristics; in some of the countries, there are shortages which limit access to several specialities (surgery, clinical oncology and radiotherapy) in some regions.

**Objective:**

After a systematic review of the most recent literature regarding the management of BC during the COVID-19 pandemic, the main objective is to understand the position of the different Latin American Societies of Mastology in terms of available alternatives for the treatment of BC.

**Methods:**

After carrying out a comprehensive and exhaustive search of the most recent guides on the management of BC during the COVID-19 pandemic, the board members of the Latin American Federation of Mastology invited, via email, different specialists, all experts in BC care, to complete an anonymous survey online.

The survey was distributed between 30 and 10 May 2020. The survey included 27 questions on four topics: demographic information, consultations, imaging and treatment of BC.

The questionnaire was sent and then distributed to various health specialists including breast surgeons, clinical oncologists, radiation oncologists and radiologists via the Presidents of the different Latin American Societies of Mastology in 18 countries. The results are summarised as tallies based on the number of responses to each question.

**Results:**

A total of 499 responses were received. The majority of the respondents were males (275 (55.11%)); 290 participants were over 45 years (58.11%).

The questionnaire presented those surveyed with three possible answers (agree, disagree and neither agree nor disagree). The results reflect that there was consensus in the majority of situations presented. Only seven questions revealed disagreement among those responding. The results are presented as recommendations.

**Conclusion:**

The management of patients with BC presents unique challenges during the current world health situation produced by COVID-19 pandemic. Breast care specialists (surgical oncologists, breast care clinicians, clinical oncologists, radiation oncologists and radiologists) from 18 countries in Central and South America submitted through their responses and recommendations for the treatment of BC during the COVID-19 pandemic.

## Introduction

The COVID-19 pandemic has motivated Latin American specialists dedicated to the diagnosis and treatment of breast cancer (BC) to suggest alternatives in the management of the care of women who suffer from this disease and then to collect information in the form of a questionnaire. This article makes recommendations regarding the management of consultations, diagnostic imaging and treatments.

The facts:

1. Management of diseases of the breast and, particularly, patients with BC presents as a challenge during the current world health situation produced by the COVID-19 pandemic.
2. There are alternative treatments to ration surgical resources and reduce possible transmission of infection from doctors to patients and vice versa.
3. The Latin American recommendations supplied for the treatment of patients with BC and non-invasive or benign lesions during the COVID-19 pandemic are based on the opinion of the great cohort of breast care specialists surveyed.

The COVID-19 pandemic has challenged health services worldwide. As of 13 May 2020, 4,276,959 people had been diagnosed with COVID SARS-2 infection, and 294,671 of those had died due to complications associated with this infection [1].

**Figure 1.**
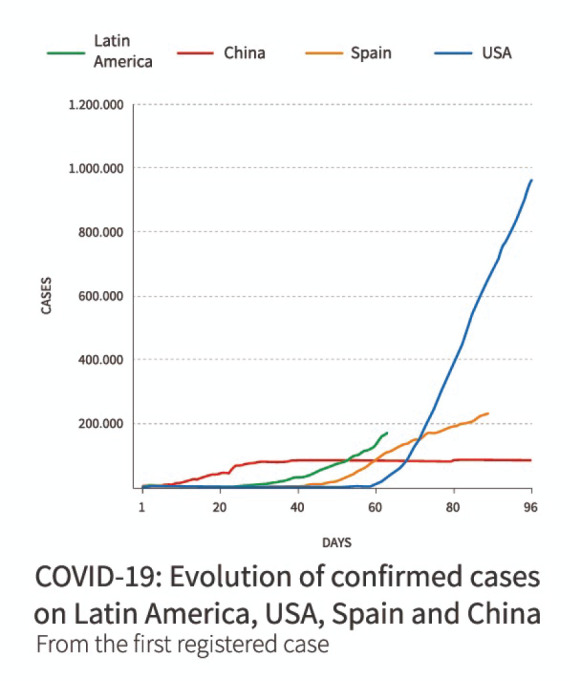
COVID-19: Progression of confirmed cases in Latin America, USA, Spain and China graph. Adapted from BBC News Mundo. Coronavirus en América Latina: 7 gráficos para entender el avance de la pandemia de COVID-19 en la región. BBC. 2020 [cited 2020 Apr 27]. Available at: https://www.bbc.com/mundo/noticias-america-latina-52405371.

In Latin America (LATAM) on this same date, the number of confirmed cases were 1,781,692, and the confirmed deaths were 106,504 [2] (Figure 1).

The care and practice of breast health will also be affected by this health crisis since new cases of BC will continue to be diagnosed during the pandemic, and there will be others whose treatments will be interrupted (surgeries, chemotherapies and radiotherapies).

Patients with this pathology require the best treatment options, in accordance with local and national resources whilst attempting to minimise the risk of exposure to the virus. Specialists in cancer care should be aware that oncology patients could have a greater risk of contracting the coronavirus infection, and therefore, it could have fatal results; moreover, patients could also worsen and die from cancer if they are not treated adequately [3].

The treatment of BC has been one of the most debated diseases, since its progression cannot be completely stopped, but it can certainly be delayed in some of the cases.

As the coronavirus pandemic spreads, many societies have published their opinions and recommendations on the steps and strategies to implement for the management of different illnesses during the crisis. We carried out a comprehensive and exhaustive search of the most recent, relevant reports on the management of BC during the coronavirus pandemic [4–6], which have been published online, bearing in mind that only recommendations based on expert opinion are included, not those trying to change cancer treatment protocols. All these are with the desire to offer help to overcome the challenge of adapting measures to each regional reality for health in particular. Until now, there are no data about the opinions of oncologists, surgeons, radiation oncologists and radiologists from LATAM on the strategies suggested in these publications neither has a statement as a Latin American group been released on the recommendations which we can give to the doctors and patients.

LATAM has several unique characteristics compared with other areas of the world. In addition, within the countries, there are individual difficulties related, for example, to the limited coverage of medical care, lack of funds and human resources and limited access to surgery, radiotherapy and chemotherapy [7]. The waiting times for patients with BC are prolonged, above all in the developing countries of the region; many centres do not have the comprehensive infrastructure to carry out adequate treatment. There are hospitals, where the waiting time for radiotherapy can be months.

The main objectives of this study were: (1) to find out, through a survey of breast care service specialists in LATAM, the acceptance rate of the adaptations adopted regarding treatment, imaging and consultation methods in females diagnosed with BC during the pandemic and (2) to use the consensus obtained, after reviewing the questionnaire, to make recommendations regarding prioritising care of women who suffer from this disease.

## Methods

The board members of the Latin American Federation of Mastology (FLAM), faced with the health problem the world’s population, are experiencing and relying on various specialists in the management of BC within their respective Mastology Societies, decided to send a survey to the presidents or representatives of each of the societies (Appendix I). In turn, the survey would be distributed amongst the specialists of their respective countries to then be able to establish how to manage different BC scenarios in the context of the COVID-19 pandemic.

To reduce the selection bias, the presidents of the different member societies of FLAM were requested to only send the questionnaires to BC specialists (Table 1).

The questionnaire included demographic characteristics of the respondent (gender and age range) and specific considerations such as the state of the respondent’s hospital, consultations, breast imaging and treatments for patients with BC (surgery, chemotherapy and radiotherapy).

Twenty-seven questions were posed with three possible answers (agree/disagree/neither agree nor disagree) (Appendix II).

The survey was designed using a link to the Google® Forms platform, which was approved by the directors of the federation. On 30 April 2020, it was distributed, via email, to the 18 presidents of the different Mastology Societies in LATAM, who had previously agreed to distribute it online amongst their associates. Reminders were sent out every 3 days via the FLAM group, created on WhatsApp, and via email. A deadline was then established for its dissemination, until 18:00 on Sunday, 10 May 2020, at which point the survey was closed.

Each link to the questionnaire was sent with a message outlining the objectives of the study, data protection feature and confidentiality.

In all the countries, participation was voluntary, without any financial incentive. The survey was available for 10 days; only one questionnaire was accepted per participant, and the responses were exported to an Excel spreadsheet provided using the same platform. Only the principal researcher had access to the results.

## Results

### Demographic characteristics of the survey participants

**Figure 2.**
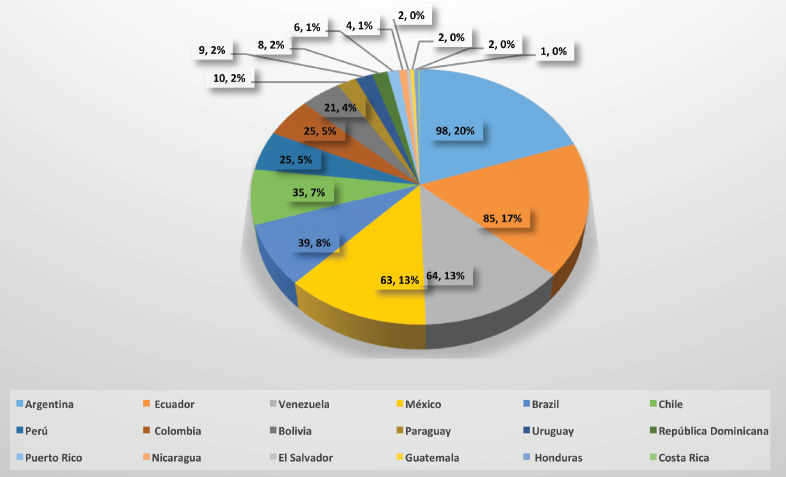
Participating countries and their responses.

On 10th May, 499 questionnaires were received from specialists in the treatment of BC, from members of 18 countries in LATAM. All the countries had at least one response from a representative of its society. The country with the greatest number of responses was Argentina (20%), followed by Ecuador (17%), Venezuela (13%), Mexico (13%), Brazil (8%), Chile (7%), Peru and Colombia (5%), Bolivia (4%) and the rest of the countries (9) between 0.2% and 3% (Figure 2).

**Figure 3.**
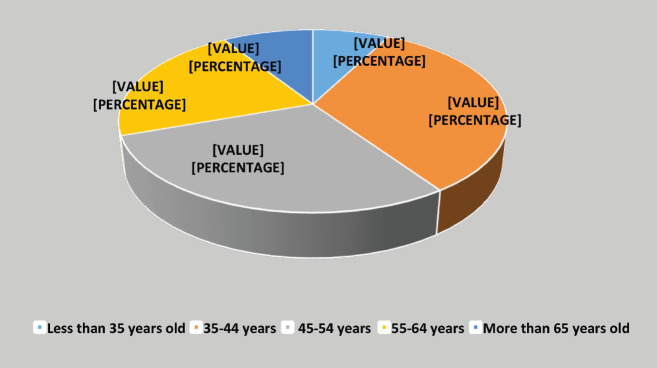
Sociodemographic characteristics of the specialist (AGE).

The majority of the surveys were responded by male BC specialists with 275 surveys (55%), and 224 were females (45%). Two hundred and ninety participants were over 45 years (58%), and 209 were under 45 (42%) (Figure 3).

### About consultations

**Figure 4.**
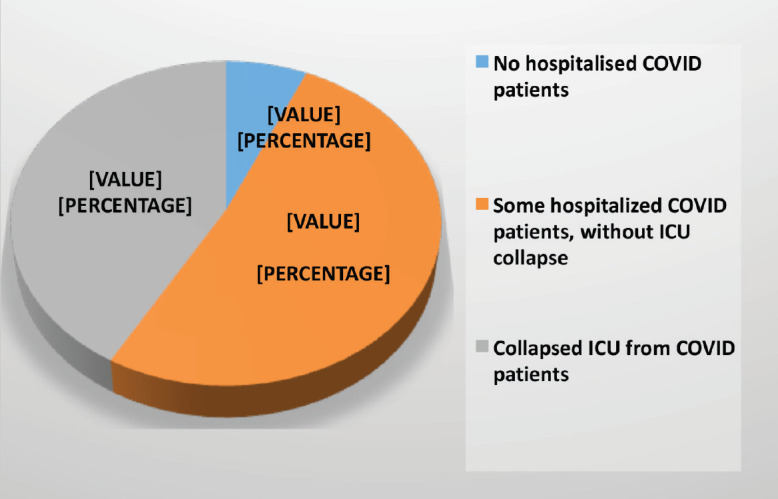
State of the hospital of the respondents during the COVID-19 pandemic.

Regarding the state of the hospitals during the time of pandemic, 6% (33) responded that they did not have any patients infected with COVID, 52% (258) had some patients hospitalised with COVID, but intensive care units (ITUs) were not at a standstill and theatres were available for any procedure; 42% (208) submitted that, in their hospitals, ITU and theatres had been brought to a standstill due to the pandemic (Figure 4).

**Figure 5.**
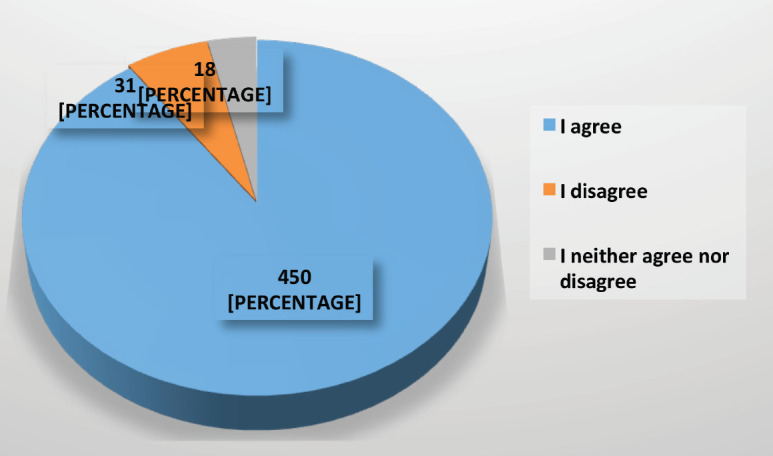
Agreement on the necessity that the assessment of a patient with a recent diagnosis of cancer should be in person during the COVID-19 pandemic.

90% of respondents were in agreement that the assessment of a patient with a recent, histologically confirmed, diagnosis of BC should be in person (Figure 5).

**Figure 6.**
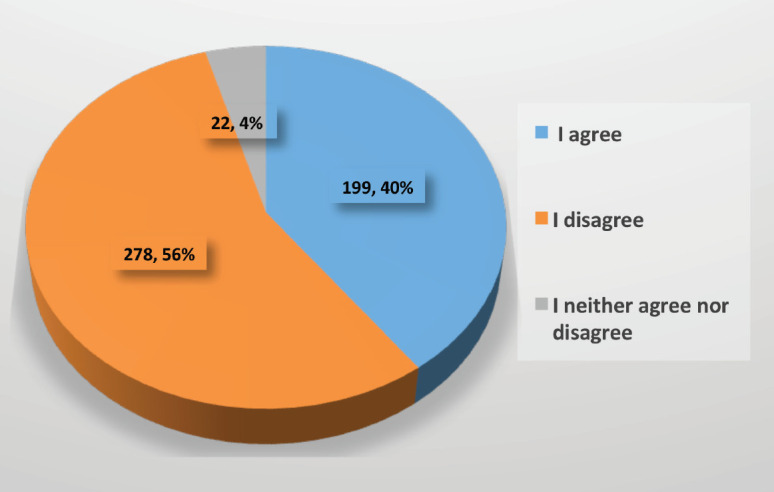
Type of consultation for postoperative assessment and revision of anatomical pathology.

**Figure 7.**
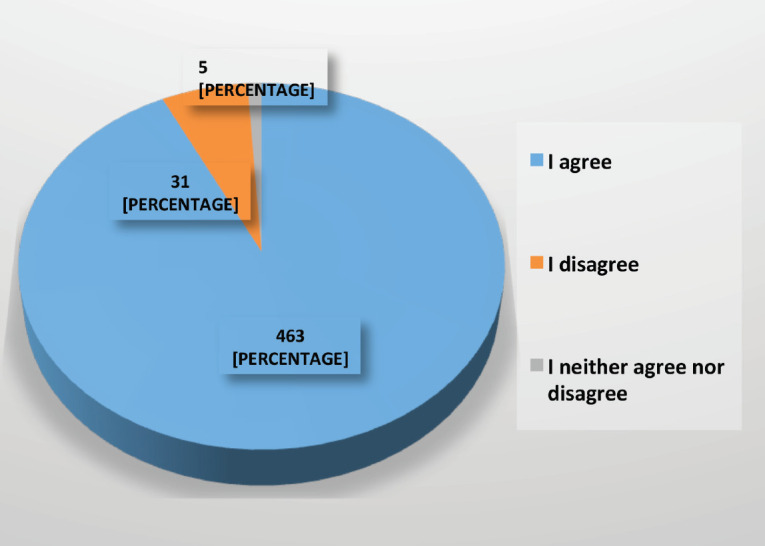
Agreement on follow-up of asymptomatic patients, post-treatment by tele-consultation during the COVID-19 pandemic.

56% of participants did not agree that the consultation for postoperative assessment and examination of their anatomical pathology should be in person (Figure 6). 93% agreed that teleconsultation, video call or email could be used to follow up patients’ post-treatment, investigating any symptoms which could raise suspicion of relapse and whether these were present to arrange an appointment for a physical examination and tests to rule out tumour recurrence (Figure 7).

In the case of not presenting these symptoms, 92% considered scheduling the next appointment for at least 3 months after the pandemic is controlled.

Regarding teleconsultations, 85% considered that they should be carried out by the treating oncology surgeon from the institution.

Fifty-eight participants suggested that other professionals could help with this function: clinical oncologists, medical residents, gynaecologists with knowledge of breast care, radiation oncologists, family doctors, nurses and general practitioners.

If it is considered that the patient should be assessed in person, 91% of the participants suggested carrying out triage on telemedicine to look for the suspected cases of COVID-19, in accordance with the current epidemiological criteria in their country.

94% of respondents agreed that for those patients in immediate life-threatening situations or who are clinically unstable and whose prognosis would be significantly altered even by a brief delay in their care, they should be assessed in person.

For patients with benign disease or follow-up consultations for surviving patients (including those with adjuvant oral medication and those who are not undergoing active treatment), 96% agreed that they could be cared for via teleconsultation or to delay the consultation until the post-pandemic period.

63% of respondents were in agreement with the use of teleconsultations or deferring consultations until the post-pandemic period for assessments on high-risk patients; 33% did not agree with this position, and 4% neither agreed nor disagreed.

### About breast imaging

Mammogram and breast ultrasounds are two fundamental examinations which aid early detection of BC, and in other patients with BC, they are used for monitoring.

Concerning the use of imaging, during the pandemic, for patients with an abnormal mammogram, suspicion of cancer, or patients requiring magnetic resonance imaging (MRI), to evaluate the extent of the disease, 89% of respondents were in agreement to carry out imaging and also procedures such as Tru-Cut biopsies on BI-RADS 4 or 5 lesions. Whilst for BI-RADS 3 lesions or patients who need routine imaging, 83% were in agreement with postponing imaging until the COVID-19 pandemic is better controlled.

Regarding screening examinations, which include mammograms, ultrasound and magnetic resonance imaging, 76% were in agreement in suspending these examinations until after the pandemic, whereas in high-risk patients such as carriers of the BRCA mutation, 66% agreed to carry out screening tests if the waiting time is going to be longer than 6 months.

### Regarding treatments for patients with BC

Table 2 shows the summary of the different situations posed in the survey, the number and percentage of the responses according to the three options (agree, disagree and neither agree nor disagree) sent by the respondents and the recommendations related to the percentage of consensus or dissent on the management of breast disease during the time of the COVID-19 pandemic.

The need to minimise the use of resources in the operating theatre requires the selective deferral of surgical procedures and to evaluate patients so that initially they use an alternative treatment whenever possible. However, the level II evidence shows that delays to treatments could affect the outcomes for patients with BC; this section of this survey is about assessing up to what point BC specialists are prepared to change normal protocols, for the use of these alternative therapies, during the time of the pandemic.

**Figure 8.**
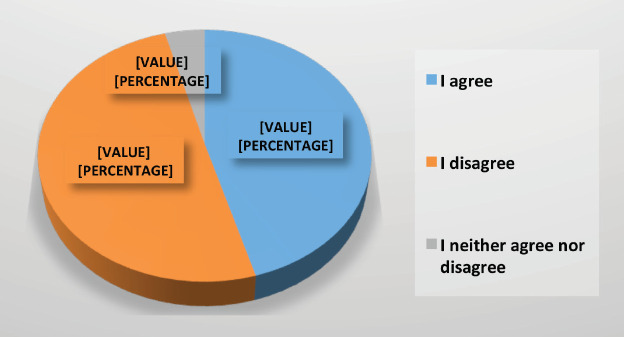
Response to the possibility of delaying surgical treatment after having finished their neoadjuvant treatment during COVID-19 pandemic.

Regarding diagnostic surgical procedures of non-suspicious lesions, 89% agreed in postponing them until after the pandemic. Only 61% of respondents believe that patients with infiltrating ductal carcinoma, initially candidates for surgery, could benefit from neoadjuvant therapies at this time. 50% of the specialists surveyed did not agree with delaying the surgical treatment of patients after having finished their neoadjuvant therapy, even knowing that a delay of up to 8 weeks would not negatively affect the outcome (Figure 8).

54% of the specialists believe that the use of local anaesthesia for the surgical treatment of BC could be a valid alternative; 33% did not agree with this stance.

The majority of guidelines and recommendations agree in proposing a diagnostic PCR test before any elective surgery; in this survey, 76% of specialists believe in the necessity of performing a COVID-19 test on every patient who is going to undergo surgery for BC.

Regarding immediate breast reconstruction with implants or tissue expanders, it has been recommended that it could be performed depending on hospital resources, limiting as much as possible the times of the operation, the risk of postoperative complications, the duration of hospitalisation and outpatient visits [8]. Autologous (flap) reconstructions should be delayed. When questioned whether all the types of deferred breast reconstructions should be postponed in the time of pandemic, 85% replied affirmatively that they were in agreement and regarding reconstruction with autologous tissue, and 79% were in agreement with deferring reconstruction until after the pandemic.

**Figure 9.**
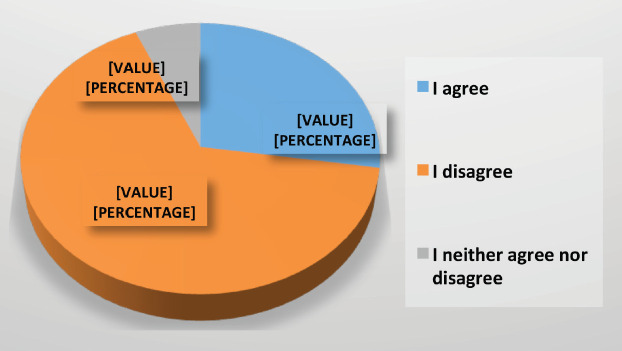
Response to the possibility of deferring re-operation on patients with positive axila or compromised borders during the COVID-19 pandemic.

Regarding the management of the axilla and the management of patients with compromised borders, 66% replied that they were in disagreement that these patients should defer re-operation until after the pandemic (Figure 9).

Regarding the surgical treatment of ductal carcinoma *in situ* (DCIS), 55% were in agreement with deferring surgery until after the pandemic, 38% disagree with postponing surgery and 7% neither agree nor disagree.

86% of respondents agree that patients elected for conservative surgery should have their surgery despite the pandemic, always taking the precautionary measures described for COVID.

Concerning the question of whether early-stage, oestrogen receptor-positive (ER +) patients could receive adjuvant radiotherapy before chemotherapy, 57% were in agreement with this position, 27% disagreed and 16% neither agreed nor disagreed.

70% of respondents replied that patients with luminal A, low- or intermediate-grade tumours could defer surgery and begin their treatment with neoadjuvant hormone therapy, whereas for triple-negative patients and HER2-/Neu-positive patients, 95% of specialists answered that they agreed that they should begin (in the case of a recent diagnosis) or continue with the standard protocols of neoadjuvant or adjuvant chemotherapy, which are already in place.

**Figure 10.**
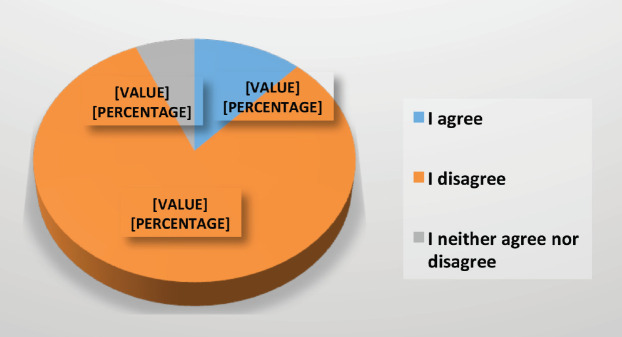
Response to the possibility of delaying treatment with radiotherapy in patients with locally advanced cancer during the COVID-19 pandemic.

The final question refers to if patients with locally advanced or inflammatory carcinoma could delay their radiotherapy treatment; 82% disagreed in delaying treatment for this group of patients (Figure 10).

## Discussion

The Royal Spanish Academy [9] defines a consensus as an ‘agreement adopted by consent amongst all members of a group’. In the branch of health sciences, to reach unanimity of all members in a group of experts is rare on matters not upheld by solid empirical evidence. Consequently, it is normally accepted that there is a consensus when the validity of a principle is shared by 80% or 90% of the experts; a majority is referred to when the validity of a principle is accepted by a minimum of two-thirds of the panel.

During this world health crisis, we have to prioritise the care of patients with BC. For this, it is necessary to consider some important factors, for example, the state of the hospital regarding the pandemic, it is also essential that administrators and professionals should be aware of local conditions and the hospital resources on which they rely, such as the availability of equipment, ITU beds and hospitalisation, to be aware of the risk of infection doctor–patient and vice versa.

The COVID-19 pandemic poses unprecedented challenges for patients, doctors and healthcare systems. In all areas of medicine, doctors are responding and adapting patient care, to minimise the risk of exposure and to preserve resources.

To provide preliminary guidance on the prioritisation and treatment of BC during this COVID-19 outbreak, we gathered together representatives from the different Latin American Mastology Societies to formulate, using a survey, the experts’ opinion. The objective of this study was to prioritise the patient scenarios according to the urgency of treatment and to make recommendations based on these priorities within each speciality. This is not a formal consensus.

On a Latin American level, few societies have sent recommendations on this subject. This is a survey reported between clinical oncologists, radiation oncologists, surgeons and medical radiologists who practice breast care in LATAM; the resulting document has the objective of making recommendations, which will serve, to support the care of patients with BC during the COVID-19 pandemic.

Publications such as COVID-19 ‘*Guidelines for Triage of BC Patients*’ [10] set out that depending on how urgent the medical consultation might be, certain patients could be evaluated via telemedicine, and others would need to be assessed in person. In LATAM, access to tools such as telemedicine by the patient and often by the specialist can be limited. Even having access to telemedicine, often the technological skills of the patient are not sufficient to carry out a consultation using this medium; this could cause the loss of follow-up and, as a consequence, has negative results for their treatment. Telemedicine could be an excellent tool to help patients with BC [11] who, in addition, have direct access and know how to use this technology.

Some studies have demonstrated the usefulness of telemedicine [12], and some guides have recommended telemedicine in this period of COVID-19 for the care of patients with BC, with the aim of reducing contact and risk of viral transmission between doctors and patients; this could be a modality used with patients who suffer from confirmed benign diseases or patients with malignant disease needing routine follow-up. These patients should delay their face-to-face consultations until after the pandemic; on the contrary, patients attending for their first consultation, with a diagnosis of cancer for whom a delay in beginning treatment, could affect the illness progression [13], patients with treatment complications, and patients undergoing treatment (be it chemotherapy or radiotherapy) could be seen in person straight after a teleconsultation. Regarding imaging for screening (mammograms, ultrasounds and magnetic resonance imaging), they could be suspended, without risk, until after the pandemic; healthy patients might be afraid or be reluctant to attend their imaging appointments under the current model, and it is likely that their participation in the early detection of BC will be reduced. On this point, an exception could be made for young patients, with a family history and genetic predisposition, to develop the disease (BRCA 1 and BRCA 2 mutation) if the waiting time exceeds 6 months [14].

Occasionally, urgent breast imaging is required: breast abscesses, haematomas or infected seroma needing drainage, clinical suspicion of inflammatory or locally advanced cancer and suspicion of BC in pregnancy [15, 16].

In the presence of mammographic suspicious lesions which involve the need to obtain a histological diagnosis (BI-RADS 4 and 5) or the need for MRI, to assess the response to neoadjuvant chemotherapy, imaging should not be delayed if hospital and staffing conditions allow; lesions classified as BI-RADS 3 can wait until after the pandemic [17].

Regarding surgical delays during the COVID-19 pandemic, fortunately, patients with BC generally do not require surgery immediately. However, we must take into consideration the waiting time involved.

For example, in Ecuador, the average waiting time for surgery in a patient with a recent BC diagnosis is approximately 2–3 months.

The extended delay could mean that, after the pandemic is controlled, no doubt the cases diagnosed during the pandemic will have accumulated along with new cases. This could bring operating theatres to a standstill and newly impact, in a negative way, the expected results in these patients. Similar situations could occur in imaging, chemotherapy and radiotherapy. BC specialists should work in conjunction with hospital administration, imaging centres and other related parties for finding a way to avoid the saturation of services once the pandemic is better controlled.

The decision to have a patient with BC undergoing surgery will be dependent on the current, national and institutional regulations, as well as on each phase of the pandemic, and of course, on the availability of resources such as the need to test all patients for coronavirus (availability of PCR and/or rapid tests), decisions regarding the urgency of the surgery and the availability of adequate personal protective equipment for surgical staff.

Given the need to minimise the use of resources in operating theatres, surgical procedures must be selectively delayed, and patients must be assessed for the use of initial alternative therapies whenever possible. However, level II evidence shows that delays in surgery can affect the results in patients with BC [14, 18].

The survey had 27 questions concerning the breast care specialism. In the majority of the responses, a consensus was reached [16]. In four cases, there was a majority, and in seven cases, there was disagreement.

Regarding the questions about the treatments of patients with BC, 6.9% (average) neither agreed nor disagreed with the question posed; 93.1% replied as being in agreement or disagreement.

Of all the questions included, there are some that show disagreement amongst respondents; some studies have explored the impact of a break between the end of the neoadjuvant chemotherapy until the time of surgery in patients with BC. These have shown that breaks of up to 8 weeks had equivalent results regarding global survival (GS) and survival free from locoregional recurrence [19, 20]. In this survey, 50% of respondents were in disagreement with delaying surgery by up to 8 weeks after neoadjuvant therapy.

The current literature has widely explored the BC relapse rates in relation with the size of the resection margins. The state of the surgical margins in a quadrantectomy specimen is a prognostic factor and is evaluated by administering dye to the surface of the surgical sample and determining the microscopic distance between the tumour cell and the dyed area. The margin is negative if there is no dye in the cancerous cells and positive if there is dye. As a consequence, 20%–30% of patients who undergo conservative breast surgery require additional surgery (re-excision) after the initial quadrantectomy [21, 22]. In response to the question as to whether re-operation should be deferred, until after the pandemic, in the case of patients with compromised margins, 66% disagreed about delaying the procedure.

The conventional treatment of DCIS is wide local excision, often followed by radiotherapy or mastectomy, and possibly hormonal therapy. The results in patients with DCIS treated with these conventional therapies are excellent, but the best way of managing DCIS is still under debate [23].

In patients with low-grade and slow-growing DCIS, active monitoring could be an alternative, after vacuum-assisted biopsies of microcalcifications. On the question about the possibility of postponing surgical treatment in patients with DCIS until after the pandemic, only 55% (277) agreed about waiting for surgery, whereas 38% disagreed.

After neoadjuvant chemotherapy and surgery (mastectomy), total radiotherapy will improve both local control and survival for patients with clinical T3 tumours or stage III–IV disease (ipsilateral supraclavicular lymph node) and patients with four or more positive lymph nodes. Radiotherapy should be considered for these patients, regardless of their response to initial chemotherapy [24].

It has been demonstrated that a delay in the time starting adjuvant chemotherapy significantly influences survival outcomes. However, the clinical impact of delaying post-operative radiotherapy is not very clear since the data have not been conclusive [25, 26]. In particular, no study specifically evaluated the neoadjuvant scenario, in which patients have high-risk disease and also had a considerably long interval without treatment before starting post-operative radiotherapy, to permit the definitive surgery. In theory, these patients could have an even greater risk of delays.

A study by Silva *et al* [27] showed that radiotherapy begun up to 8 weeks after surgery in patients with locally advanced disease, who had previously undergone neoadjuvant chemotherapy, was associated with a better global survival and survival free from disease.

For the question, if you believe that patients with locally advanced or inflammatory BC could delay their radiotherapy treatment until after the pandemic, 82% (408) declared themselves in disagreement with this statement.

It poses the possibility of the use of local and/or regional anaesthetic as a valid alternative for the surgical treatment of BC, which would be an advantage during this pandemic. This model would help reduce the risks of general anaesthesia, postoperative pain, nausea, vomiting and above all the duration of the hospital stay, lower risk of infection for the patient and doctor and a saving in economic resources [28, 29]. For this question, 54% (269) of the specialists were in agreement over the possibility of using local and regional anaesthesia for BC surgery, during this period of COVID-19.

## Conclusion

This study of LATAM gathered together the opinion of a great cohort of breast care specialists (surgeons, clinical oncologists, radiation oncologists, radiologists and others) on how to manage BC during this serious situation for the world’s health, which is also affecting the continent. The reality is different compared to other continents since, in the countries, treatments are already delayed and, in this time of pandemic, the problem is increased. Consequently, we can conclude that despite the pandemic, it is necessary to try to dedicate adequate attention to the patients, supported in some cases with teleconsultations, and to know how to classify those patients who merit a consultation in person; with this, we will reduce the risk of infection from patient to doctor.

Regarding the imaging studies, they could all be delayed with exception to the patients who need them due to a suspicious lesion and a biopsy or those with high genetic risk, where the wait should not be prolonged, or those who need imaging to assess persistence or multicentricity during treatment.

Regarding treatment, the majority of respondents disagreed in delaying so many surgeries, chemotherapy treatments, which are adjuvant or neoadjuvant, and radiotherapy. However, each case should be discussed individually within the multidisciplinary team, assessing that the potential damage could cause a delay in treatments. The majority were in agreement in delaying breast reconstructions of all the deferrals.

The COVID-19 pandemic has presented unique challenges and learning opportunities for BC specialists.

The evolution of this pandemic is still uncertain, and the general population should stay alert to reduce its impact.

The policies will carry on changing, depending on the momentum that each region experiences, from the response of its authorities, and the attitude of its doctors and patients towards the pandemic. These new conditions could change some of the recommendations.

## Funding

This study was not supported by any person or institution.

## Conflicts of interest

The authors have not declared any conflicts of interest.

## Authors’ contributions

All authors contributed equally to this research project.

## Figures and Tables

**Table 1.** Society/number of members/percentage of responses per country.

Country	Number of members	Numbers of responses per country (%)
Argentina	743	98 (13.18)
Ecuador	90	85 (94.4)
Venezuela	350	64 (18.28)
Mexico	800	63 (7.87)
Brazil	2000	39 (1.95)
Chile	140	35 (25)
Peru	143	25 (17.4)
Colombia	144	25 (17.36)
Bolivia	75	21 (28)
Paraguay	46	10 (21.73)
Uruguay	106	9 (8.49)

Societies that reported the number of members.

**Table 2.** Summary of the different situations posed in the survey, the number and percentage of the responses according to the three options (agree, disagree, and neither agree nor disagree) sent by the respondents, and the recommendations related to the percentage of consensus or dissent on the management of breast disease during this time of the COVID-19 pandemic.

Description of situations posed	Responses of those surveyed according to the options posed (499 responses)			Recommendations based on the survey	Consensus/no consensus/majority
		Agree Responses (%)	Disagree Responses (%)	Neither agree nor disagree Responses (%)	
Consultation	Should a face-to-face consultation be carried out for patients with a histologically confirmed diagnosis of BC?	450 (90 )	31 (6)	18 (4)	Consensus on the need for a face-to-face consultation
	Should appointments for revision of reports or follow-up of patients post-treatment be carried out by teleconsultation?	463 (93)	31 (6)	5 (1)	There is a consensus that the revision of reports or post-treatment follow-up should be carried out by teleconsultation
	Should the postoperative assessment for the revision of anatomical pathology be in person?	199 (40)	278 (56)	22 (4)	There is no consensus that the assessment of anatomical pathology should be in person
	Should it be the specialist who carries out the teleconsultation?	424 (85)	58 (12)	17 (3)	Consensus: the Surgical Oncologist or Breast Care specialist
	Should a triage be carried out by teleconsultation in the case of needing a face-to-face consultation	457 (91)	33 (7)	9 (2)	There is consensus in carrying out triage via telemedicine to search for suspected cases of COVID-19, in accordance with the current epidemiological criteria of their countries
	Patients whose condition puts their life in immediate danger are clinically unstable and who even a brief delay in care would significantly alter their prognosis should be assessed in person	470 (94)	23 (5)	6 (1)	Consensus on the need for a face-to-face consultation
	Should follow-up for patients with benign disease or follow-up consultations of surviving patients be delayed until after the pandemic?	480 (96)	16 (3)	3 (1)	Consensus in delaying the consultation until after the pandemic, they should be seen using tele-consultation
Imaging	Lesions BI-RADS 3 or patients who need routine imaging could be delayed until after the pandemic	412 (83)	67 (13)	20 (4)	Consensus in postponing until after the pandemic
	During the pandemic, diagnostic imaging should be carried out for abnormal mammographies, suspicions of cancer, biopsies of lesions BI-RADS 4, patients who require an MRI to evaluate the extent of disease	445 (89)	41 (8)	13 (3)	Consensus in not postponing imaging, nor delaying taking biopsies on BI-RADS IV lesions
Treatments	Regarding diagnostic surgical procedures for non-suspicious lesions, should they be delayed until after the pandemic?	444 (89)	49 (10)	6 (1)	
	Regarding the delay of a patient’s surgical treatment after having finished their neoadjuvant treatment	227 (46)	251 (50)	21 (4)	There was no consensus in delaying surgical treatment after finishing neoadjuvant treatment
	Regarding the use of local anaesthesia as an alternative in surgical treatment during the pandemic	269 (54)	166 (33)	64 (13)	There was no consensus for the use of local anaesthesia in the surgical treatment of BC during the pandemic
	Regarding postponing surgical treatment in patients with DCIS until after the pandemic	277 (55)	188 (38)	34 (7)	There was no consensus on the possibility of delaying surgery in patients with DCIS
	Regarding patients operated on with early-stage, ER-positive tumours, could they receive radiotherapy treatment before adjuvant chemotherapy during the pandemic?	284 (57)	136 (27)	79 (16)	There was no consensus over the possibility that patients with early-stage, ER-positive BC could receive radiotherapy before chemotherapy during the pandemic
	A COVID test should be carried out on every patient who is going to undergo surgery for BC	381 (76)	86 (17)	32 (7)	The majority answered that a COVID test should be performed
	All delayed breast reconstructions (DBR) should be postponed until after the pandemic	424 (85)	64 (13)	11 (2)	Consensus that DBR should be postponed until after the pandemic
	Should all breast reconstructions (BR) with autologous tissues be postponed until after the pandemic?	393 (79)	88 (18)	18 (3)	Consensus that BR with autologous tissues should be postponed until after the pandemic
	Should the surgery for compromised microscopic margins and axilla staging be deferred until after the pandemic?	330 (66)	137 (28)	32 (6)	The was no consensus in postponing the re-operation on patients with compromised margins and axilla staging
	Should patients chosen for conservative surgery have their surgical treatment regardless of the pandemic?	428 (86)	49 (10)	22 (4)	Consensus in not postponing surgery
	Should triple-negative and HER2-/Neu-positive patients continue treatments already underway?	475 (95)	11 (2)	13 (3)	Consensus in not stopping nor postponing treatments underway in HER2-/Neu-positive and triple-negative patients
	Patients with locally advanced or inflammatory BC could postpone their treatment until after the pandemic?	59 (12)	408 (82)	32 (6)	There is no consensus in delaying treatment in patients with locally advanced BC

## Appendix I

**Table A1.** Societies surveyed and their president or representative.

Societies surveyed	President or representative
Argentinian Society of Mastology (SAM)	Dr. Eduardo Gonzalez
Ecuadorian Society of Breast Pathology (SEPAM)	Dr. Antonio Jurado B.
Bolivian Society of Mastology (SBM)	Dr. Roger Corrales
Colombian Association of Mastology (ACM)	Dr. Luis Fernando Viaña
Uruguayan Society of Mastology (SUM)	Dr. Guillermo Laviña
Chilean Society of Mastology	Dr. Paula Escobar
Mexican Association of Mastology (AMM)	Dr. Víctor Manuel Pérez
Brazilian Society of Mastology (SBM)	Dr. Vilmar Marques de Oliveira
Dominican Society of Surgery	Dr. Miguel Oller
Peruvian Society of Mastology	Dr. José Galarreta
Paraguayan Society of Mastology	Dr. Valeria Sanabria
Venezuelan Society of Mastology	Dr. Jorge Pérez Fuentes
Costa Rican Society of Mastology	Dr. Gonzalo Vargas Chacón
Nicaraguan Mastology Association	Dr. Verónica Avilés
Honduran Mastology Society	Dr. Lía Bueso de Castellanos
Puerto Rican Society of Mastology	Dr. Bolívar Arboleda Osorio
Guatemalan Society of Mastology	Dr. Herbert Castillo
El Salvador Society of Mastology	Dr. Claudia Figueroa

## Appendix II

### Survey

#### Sociodemographic characteristics of the specialist

Age *

- Younger than 35
- 35–44
- 45–54
-  55–64
- >65

Gender *

- Female
- Male

COUNTRY:

- Argentina
- Chile
- Brazil
- Uruguay
- Paraguay
- Ecuador
- Colombia
- Peru
- Venezuela
- Bolivia
- Mexico
- El Salvador
- Honduras
- Guatemala
- Costa Rica
- Puerto Rico
- Nicaragua
- Dominican Republic

#### (A) CONSULTATIONS

1. What is the current state of your hospital?
  - Hospital with ITU at a standstill due to patients with COVID
  - Some patients hospitalised with COVID, ITU still functioning and operating theatres available.
  - Hospital with ITU at a standstill due to patients with COVID
2. Do you consider that patients with a high suspicion of or with a histologically confirmed diagnosis of BC should be assessed, for the first time, in person? *
  - Agree
  - Disagree
  - Neither agree nor disagree
3. Do you consider that postoperative surgical BC patients should be assessed in person for revision of their anatomical pathology? *
  - Agree
  - Disagree
  - Neither agree nor disagree
4. Do you believe that for patients with a diagnosis of BC, post-treatment follow up should be conducted via teleconsultation, to investigate any symptoms which could be suspicious of recurring disease, and in the case of presenting them, arrange an appointment for a physical examination and tests to rule out tumour recurrence? *
  - Agree
  - Disagree
  - Neither agree nor disagree
5. Do you believe that for patients with a diagnosis of BC, post-treatment follow-up should be conducted via teleconsultation, to investigate any symptoms which could be suspicious of recurring disease, and in the case of not presenting any, schedule a further follow-up appointment in the following 3 months after the pandemic is controlled?
  - Agree
  - Disagree
  - Neither agree nor disagree
6. Do you believe that the teleconsultation should be carried out by the treating oncology surgeon from the institution? In case of disagreement which health professional do you suggest could carry out this process? *
  - Agree
  - Disagree
  - Neither agree nor disagree
  6.1 If in the previous question you answered that you disagree, please mention which other healthcare professionals should carry out the teleconsultation __________________________________________
7. If you consider that the patient should be assessed in person, do you suggest that patients should be triaged using teleconsultation to look for the suspected cases of COVID-19, according to the current epidemiological criteria? *
  - Agree
  - Disagree
  - Neither agree nor disagree
8. Do you think that patients whose condition puts their life in immediate danger, for example, clinically unstable post-operative patients, those with possible medical oncology emergencies (for example neutropenic fever, untreatable pain) and those who even a brief delay would significantly affect the patient’s prognosis should be evaluated in person?
  - Agree
  - Disagree
  - Neither agree nor disagree
9. Do you believe that patients who present for routine follow-up for benign or malignant disease (including those on adjuvant oral medication and those who are not undergoing active treatment) and consultations for surviving patients can be seen remotely or have their consultations delayed until the post-pandemic period?
  - Agree
  - Disagree
  - Neither agree nor disagree
10. Do you believe that consultations for high-risk assessments can be done remotely or delayed until the post-pandemic period?
  - Agree
  - Disagree
  - Neither agree nor disagree

#### (B) BREAST IMAGING

1. Do you believe that during the pandemic, diagnostic imaging should be performed for abnormal mammograms, or for suspicious breast symptoms, BI-RADs 4 or 5 lesion biopsies, and breast MRIs to evaluate the extent of the disease or evaluation after neoadjuvant chemotherapy?
  - Agree
  - Disagree
  - Neither agree nor disagree
2. Do you believe that patients with category 3 BI-RADs who return for a short-term follow-up diagnostic mammogram and/or a routine ultrasound scan and a breast examination should be postponed until the end of the COVID-19 pandemic?
  - Agree
  - Disagree
  - Neither agree nor disagree
3. Do you believe that all screening tests including mammograms, ultrasound and magnetic resonance imaging should be suspended until the period after COVID-19?
  - Agree
  - Disagree
  - Neither agree nor disagree
4. Do you believe that patients carrying the BRCA mutation, under 40 years, should be considered for screening if delays greater than 6 months are expected?
  - Agree
  - Disagree
  - Neither agree nor disagree

#### C) TREATMENTS FOR PATIENTS WITH BC

1. Do you believe that diagnostic surgical procedures of non-suspicious lesions should be postponed until after the pandemic?
  - Agree
  - Disagree
  - Neither agree nor disagree
2. Do you believe that surgical candidates diagnosed with IDC could potentially benefit from neoadjuvant treatments?
  - Agree
  - Disagree
  - Neither agree nor disagree
3. Do you believe that patients who complete neoadjuvant chemotherapy could delay surgical treatment until the end of the pandemic, knowing that delays in surgery of up to 8 weeks after chemotherapy do not negatively affect the results?
  - Agree
  - Disagree
  - Neither agree nor disagree
4. Do you believe that local and/or regional anaesthesia is a valid alternative for the surgical treatment of BC during the time of pandemic?
  - Agree
  - Disagree
  - Neither agree nor disagree
5. Do you believe in the necessity of requesting COVID-19 tests from every patient who is going to undergo BC surgery?
  - Agree
  - Disagree
  - Neither agree nor disagree
6. Do you believe that all types of breast reconstruction should be delayed until the pandemic has passed?
  - Agree
  - Disagree
  - Neither agree nor disagree
7. Do you believe that all immediate reconstructions with autologous tissues should be delayed until after the pandemic?
  - Agree
  - Disagree
  - Neither agree nor disagree
8. Do you believe that the re-operation of compromised microscopic margins and axilla staging should be deferred until after the pandemic?
  - Agree
  - Disagree
  - Neither agree nor disagree
9. Do you believe that a patient with a diagnosis of DCIS could postpone their surgical treatment until after the pandemic?
  - Agree
  - Disagree
  - Neither agree nor disagree
10. Do you believe that patients chosen for conservative surgery during the pandemic should have their surgical treatment regardless of the pandemic taking all the precautions described for COVID?
  - Agree
  - Disagree
  - Neither agree nor disagree
11. Do you believe, constantly assessing the situation in your region regarding COVID-19, that patients already operated on with, early-stage, ER-positive tumours could receive radiotherapy treatment before adjuvant chemotherapy during the pandemic?
  - Agree
  - Disagree
  - Neither agree nor disagree
12. Do you believe that patients with early-stage, luminale A, intermediate or low-grade tumours could defer surgery and receive neoadjuvant endocrine therapy until after the pandemic?
  - Agree
  - Disagree
  - Neither agree nor disagree
13. Do you believe that patients with triple-negative or HER2-/Neu-positive BC should commence (in the case of a recent diagnosis) or continue with the standard protocols of neoadjuvant or adjuvant chemotherapy which are already in place?
  - Agree
  - Disagree
  - Neither agree nor disagree
14. Do you believe that patients with locally advanced or inflammatory BC could delay their radiotherapy treatment until after the pandemic?
  - Agree
  - Disagree
  - Neither agree nor disagree
